# Reversible Acute Kidney Injury Associated with Sildenafil Overdose

**DOI:** 10.7759/cureus.3322

**Published:** 2018-09-17

**Authors:** Baoqiong Liu, Lingbin Meng, Xuan Guan, Lu Gao, Joshua Trabin

**Affiliations:** 1 Internal Medicine, Florida Hospital, Orlando, USA

**Keywords:** acute tubular necrosis, sildenafil, overdose

## Abstract

Sildenafil is a phosphodiesterase type 5 inhibitor that is approved to treat erectile dysfunction and pulmonary hypertension. Generally, sildenafil is safe, with mild side effects. Here, we report a case of acute kidney injury caused by a sildenafil overdose. A 67-year-old man took 400 mg of sildenafil for erectile dysfunction. The patient was found to have acute kidney injury from acute tubular necrosis during hospitalization with a peak serum creatinine of 5.07 mg/dL though his renal function recovered with supportive care.

## Introduction

Sildenafil is a widely used medication for the treatment of erectile dysfunction and pulmonary hypertension [1-2]. Sildenafil acts by inhibiting cyclic guanosine monophosphate (cGMP)-specific phosphodiesterase type 5 (PDE5), which is responsible for the degradation of cGMP. Increased levels of cGMP produce smooth muscle relaxation and vasodilation [3]. Sildenafil is a safe medication, with mild side effects of headaches, heartburn, and flushed skin. Rare but serious side effects include sudden-onset hearing loss and prolonged erections, which can lead to damage to the penis [4]. Here, we report a case of acute kidney injury following the use of sildenafil.

## Case presentation

A 67-year-old male with a history of chronic kidney disease (CKD) stage 2 with a baseline creatinine of 1.25 mg/dl, hypertension, diabetes, and coronary artery disease, presented to us with difficulty urinating for four hours. The patient took a total of 400 mg sildenafil between 2 pm and 6 pm on the day of admission. He had sex at around 7:30 pm. After that, he was having difficulty urinating, with clamminess and intermittent palpitations. He arrived in the emergency department at 11:55 pm. He developed hematuria after Foley catheter placement for urinary retention. Home medication included lisinopril 40 mg daily for hypertension and clopidogrel post stent placement.

At admission, his temperature was 97.5 °F, and his blood pressure was 91/62 mmHg. An examination was remarkable for an absent left testicle due to orchiectomy in childhood. Serum creatinine and blood urea nitrogen (BUN) were 1.94 mg/dl and 16 mg/dl, respectively. Urinalysis revealed albumin 3+. Urine culture was negative. Parathyroid hormone (PTH) was elevated at 304 pg/ml. The calculated fractional excretion of sodium (FeNa) was 1.4%. Ultrasound showed increased echogenicity of bilateral kidneys.

**Figure 1:**
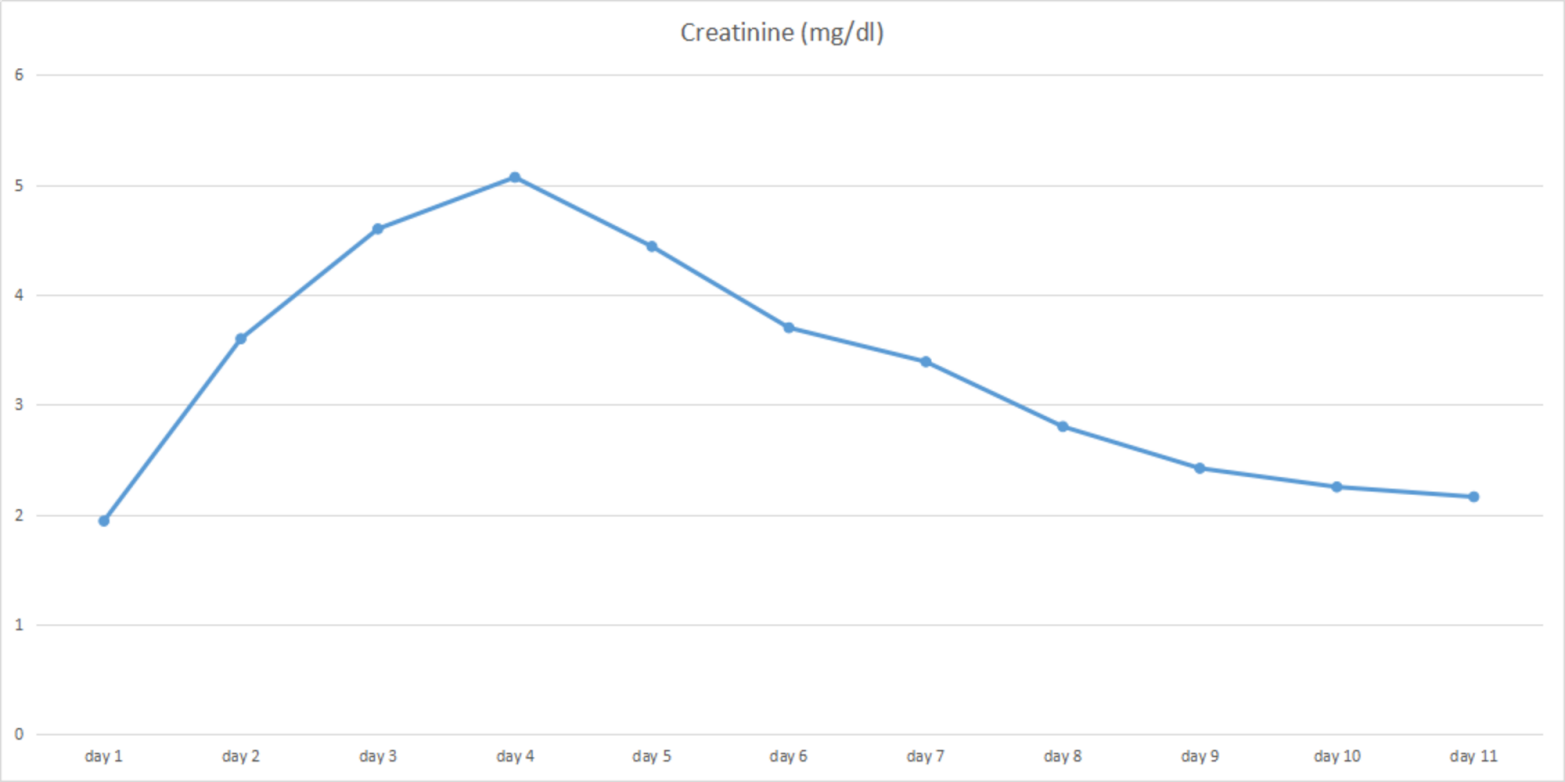
Graph illustrating trends in the serum creatinine of the patient during hospitalization

**Figure 2:**
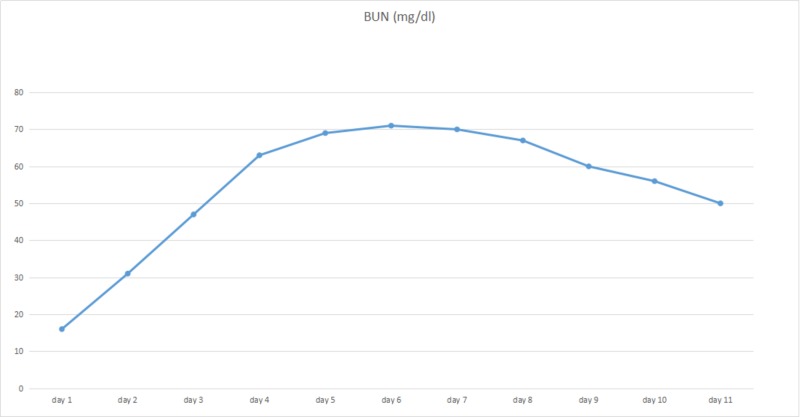
Graph illustrating trends in the blood urea nitrogen (BUN) of the patient during hospitalization

The patient was put on continuous bladder irrigation for hematuria. He received two liters of normal saline bolus in the emergency department upon presentation and was continued on intravenous normal saline at a rate of 100 ml/hr. On the second day of admission, he developed lower extremities edema. Urine output was not calculable because of continuous bladder irrigation. His creatinine was increased from 1.94 mg/dl to 3.60 mg/dl for which intravenous fluid was suspended and one dose of 40 mg intravenous furosemide was administered; however, serum creatinine continued to rise to 4.60 mg/dl and his 24-hour estimated urine output was less than 400 ml, for which nephrology service was consulted and the patient was put on normal saline intravenous infusion at a rate of 50 ml/hr, clopidogrel was discontinued due to persistent hematuria, and bladder irrigation continued. On Day 4, his serum creatinine rose to 5.07 mg/dl, intravenous (IV) fluid was then suspended and intravenous furosemide was increased to 80 mg twice daily. On Day 5, the number dropped to 4.44 mg/dl, and they continued to improve as 3.70 mg/dl on Day 6 and 3.39 mg/dl on Day 7. Peak serum creatinine was recorded at 5.07 mg/dl on Day 4 (Figure 1) while blood urea nitrogen (BUN) peaked on Day 6 at 71 mg/dl (Figure 2). On Day 8, he was in a polyuric state with a urine output of four liters. A biopsy was not performed since it would not change the management and his kidney function was improved during a short period of time. The patient was discharged with a creatinine at 2.18 mg/dl on Day 11 and he never required dialysis. Based on the clinical scenery, a diagnosis of acute kidney injury due to acute tubular necrosis (ATN) was made. Hypoperfusion of kidneys induced by sildenafil overdose was concluded as the cause of his acute tubular necrosis, even though his blood pressure measured at the emergency department and during hospitalization was within normal limits.

## Discussion

Sildenafil was approved by the Food and Drug Administration (FDA) in 1998 to treat erectile dysfunction. More than 20 million men were treated with sildenafil in its first six years on the market [5]. The recommended usual dose of sildenafil for erectile dysfunction is 50 mg once daily, one hour before sexual activity, with a maximum dose of 100 mg once daily.

To our knowledge, there has been only one case of acute tubular necrosis (ATN) associated with sildenafil reported, which is in a 39-year-old man who developed ATN after taking 200 mg sildenafil on two consecutive days [6]. Our patient took 400 mg of sildenafil in four hours and subsequently developed the hypotensive symptoms of trenching sweating and palpitations. He had a history of CKD stage 2 with a baseline creatinine of 1.25, which translates to an estimated glomerular filtration rate (GFR) of 65 to 70 mL/minute. With the microalbuminuria on urinalysis, his CKD was considered to be caused by diabetes. The patient did not take any nonsteroidal anti-inflammatory drugs (NSAIDs) or any herbal supplements nor had any exposure to iodinated contrast. Based on the clinical course, ATN from the hypoperfusion of kidneys induced by a sildenafil overdose was concluded as the cause of his acute kidney injury even though his blood pressure measured at the emergency department was within normal limits. However, there was no feasible way to know how low his blood pressure dropped to by the time he had the hypotensive symptoms at home. The patient’s adaptive responses of his kidneys were very likely impaired due to a history of hypertension and diabetes, CKD, and lisinopril use. Therefore, he was at high risk for normotensive ischemic ATN when his blood pressure was lowered below his usual levels, even though the levels remained in the normal range.

The differential diagnosis of this case mainly includes a prerenal acute kidney injury. However, this was inconsistent with FeNa >1% measured at presentation. And unlike in a prerenal acute kidney injury, the kidney function in ischemic ATN does not improve with the restoration of renal blood flow [7]. As observed in this patient, his kidney function continued to worsen in spite of receiving intravenous fluids during the first few days. Another differential diagnosis would be a postrenal acute kidney injury, as the patient had urinary retention, which was likely related to sildenafil use. But the possibility was also very low because the patient's urinary retention was temporary, which resolved soon after a Foley insertion in the emergency department. The clinical presentation of hypotensive symptoms, oliguria (urine output of <500 mL in 24 hours), and polyuria in this case, despite the absence of a pathological test, is most compatible with ATN.

ATN is a common cause of acute kidney injury in the hospital setting. It normally goes through an oliguric phase of one to two weeks followed by a diuretic phase of 10-14 days [8]. But it is possible that the oliguric phase may last less than 24 hours [9]. The diuretic phase, in which tubular function is restored, is characterized by an increase in urine volume and by a gradual decrease in blood urea nitrogen (BUN) and serum creatinine to their pre-injury levels [8]. This patient had a sudden increase in BUN and serum creatinine, which started to improve five days after onset.

## Conclusions

In conclusion, sildenafil use in patients with existing kidney disease should carry extra caution especially with overdosing, which may lead to devastating complications. The chances of ATN may be even higher if it is taken together with nitrates. Drinking plenty of fluids while taking sildenafil may prevent or decrease the severity of ATN. Once ATN has developed, the majority of treatment is supportive, with either a fluid or a diuretics challenge depending on the clinical scenarios, as well as avoiding nephrotoxin and profound hypotension.
